# Adenosine: Direct and Indirect Actions on Gastric Acid Secretion

**DOI:** 10.3389/fphys.2017.00737

**Published:** 2017-09-22

**Authors:** Rosa M. Arin, Adriana Gorostidi, Hiart Navarro-Imaz, Yuri Rueda, Olatz Fresnedo, Begoña Ochoa

**Affiliations:** Department of Physiology, Faculty of Medicine and Nursing, University of the Basque Country (UPV/EHU) Leioa, Spain

**Keywords:** extracellular adenosine, enteric nervous system, purinergic signaling, gastric acid secretion regulation, gastric mucosa

## Abstract

Composed by a molecule of adenine and a molecule of ribose, adenosine is a paradigm of recyclable nucleoside with a multiplicity of functions that occupies a privileged position in the metabolic and regulatory contexts. Adenosine is formed continuously in intracellular and extracellular locations of all tissues. Extracellular adenosine is a signaling molecule, able to modulate a vast range of physiologic responses in many cells and organs, including digestive organs. The adenosine A1, A2A, A2B, and A3 receptors are P1 purinergic receptors, G protein-coupled proteins implicated in tissue protection. This review is focused on gastric acid secretion, a process centered on the parietal cell of the stomach, which contains large amounts of H^+^/K^+^-ATPase, the proton pump responsible for proton extrusion during acid secretion. Gastric acid secretion is regulated by an extensive collection of neural stimuli and endocrine and paracrine agents, which act either directly at membrane receptors of the parietal cell or indirectly through other regulatory cells of the gastric mucosa, as well as mechanic and chemic stimuli. In this review, after briefly introducing these points, we condense the current body of knowledge about the modulating action of adenosine on the pathophysiology of gastric acid secretion and update its significance based on recent findings in gastric mucosa and parietal cells in humans and animal models.

## Introduction

Adenosine is a purine nucleoside widely found in nature. It is a component of the nucleotides ATP, ADP, AMP, of the cyclic nucleotide cAMP, of the nucleotide polymer RNA in all its forms and of the redox coenzymes NAD^+^, NADP^+^, and FAD, all of which are critic molecules for (unicellular and multicellular) life. However, in the extracellular space adenosine is a signaling molecule, able to bind to and activate four different G protein-coupled receptors (GPCRs) designated A1, A2A, A2B, and A3 (Fredholm et al., 2001a). Receptors for adenosine are expressed across species and by virtually all tissues (Burnstock, 2007; Fredholm and Verkhratsky, 2010). The gastrointestinal tract is an adenosine target but, whereas a role for adenosine in dampening intestinal inflammation is rather well established (reviewed, among others, by Ye and Rajendran, 2009; Colgan and Eltzschig, 2012; Borea et al., 2016) and the precise localization of adenosine receptors in the human small and large intestine is known (Antonioli et al., 2008; Christofi, 2008), the body of evidence concerning the impact of adenosine on one of the most important tasks of the stomach, i.e., acid secretion, is yet fragmentary and inconclusive.

The stomach fulfills important tasks in the mechanic and chemic digestion of food. The acidic gastric juice acts as a barrier against ingested pathogens and makes enzymatic digestion possible. During digestion the pH of the gastric lumen reaches values of 1–2, which requires specialized cells to produce and secrete great amounts of hydrochloric acid. Those cells are called parietal cells and are mainly located in the oxyntic mucosa. Stomach is equipped with protective measures to ensure that its mucosa is not damaged by acid. Mucous cells of the neck of gastric glands secrete protective mucus and surface cells secrete bicarbonate. That generates an alkaline mucus barrier that protects mucosal surface while gastric contents remain acidic. On the other hand, gastric acid secretion must be tightly regulated. Several cells of gastric mucosa including gastrin-secreting (G), enterochromaffin-like (ECL), and somatostain-producing (D) cells plus neuronal and mechanic stimulation participate in a regulatory network that ultimately controls acid secretion by parietal cells. An imbalance in protective mechanisms or in acid secretion regulation can lead to hyposecretion or hypersecretion of acid or diseases like gastroesophageal reflux disease, which has an estimated prevalence of about 20% in Europe and South and North America that can reach up to 33% in the Middle East (El-Serag et al., 2014).

The human stomach is organized into two functional areas: the oxyntic area (fundus and corpus or body), where most of the parietal cells reside, and the antrum or pyloric area, containing most of the gastrin-secreting G cells as well as mixed-type glands positive for both parietal and G cells (Choi et al., 2014).

After summarizing the most studied regulatory pathways of acid secretion, the current review will mainly focus on the sources and action mechanisms of extracellular adenosine. Furthermore, it puts together the body of evidence that exists about the role of adenosine in acid secretion regulation, pinpointing seemingly paradoxical actions in different study models.

## Acid secretion and its regulation

Parietal cells are highly specialized epithelial cells, with distinctive morphologic features that support their acid-secreting function. In resting state, the apical plasma membrane presents small invaginations or canaliculi that project throughout the cell interior and interconnect. Cytoplasm contains abundant membrane structures called tubulovesicles rich in H^+^/K^+^-ATPase, the proton pump responsible for proton extrusion during acid secretion (Duman et al., 2002). Tubulovesicles fuse with the apical plasma membrane upon activation of acid secretion, and so the morphology of the cell undergoes a dramatic change, with enlarged canaliculi and longer microvilli (Forte et al., 1977; Forte and Yao, 1996).

H^+^/K^+^-ATPase exchanges an intracellular hydrogen ion for an extracellular potassium ion, consuming ATP in the process. H^+^/K^+^-ATPase can generate a gradient of 6 pH units. Sustained proton extrusion requires two other ion transport processes to occur in the apical plasma membrane of parietal cells. One of them is chloride secretion, which is necessary to maintain electroneutrality during acid secretion. The precise identity of the chloride ion pathway has not been established yet, and at least three candidates are considered: cystic fibrosis conductance regulator (Sidani et al., 2007), chloride channel protein 2 (Malinowska et al., 1995; Hori et al., 2004), and solute carrier (SLC) 26A9 (Xu et al., 2008). The other one is potassium recycling, necessary to avoid luminal potassium depletion, which would impair H^+^/K^+^-ATPase activity (Heitzmann and Warth, 2007). Potassium must leak into the lumen through channels or transporters, but the exact pathway potassium takes has not been elucidated. Candidates include the voltage-gated potassium channel KCNQ1 (Heitzmann and Warth, 2007), several inward-rectifier potassium channel (K_ir_) family members (Malinowska et al., 2004; Kaufhold et al., 2008) and K-2Cl cotransporter KCC4 (Fujii et al., 2009).

Basolateral ion transport is also required in acid secretion. Those processes compensate for apically secreted ions and maintain intracellular pH by secreting bicarbonate ions to the extracellular fluid. More information on this matter can be found in other review works (Kopic et al., 2010).

**Figure 1:**
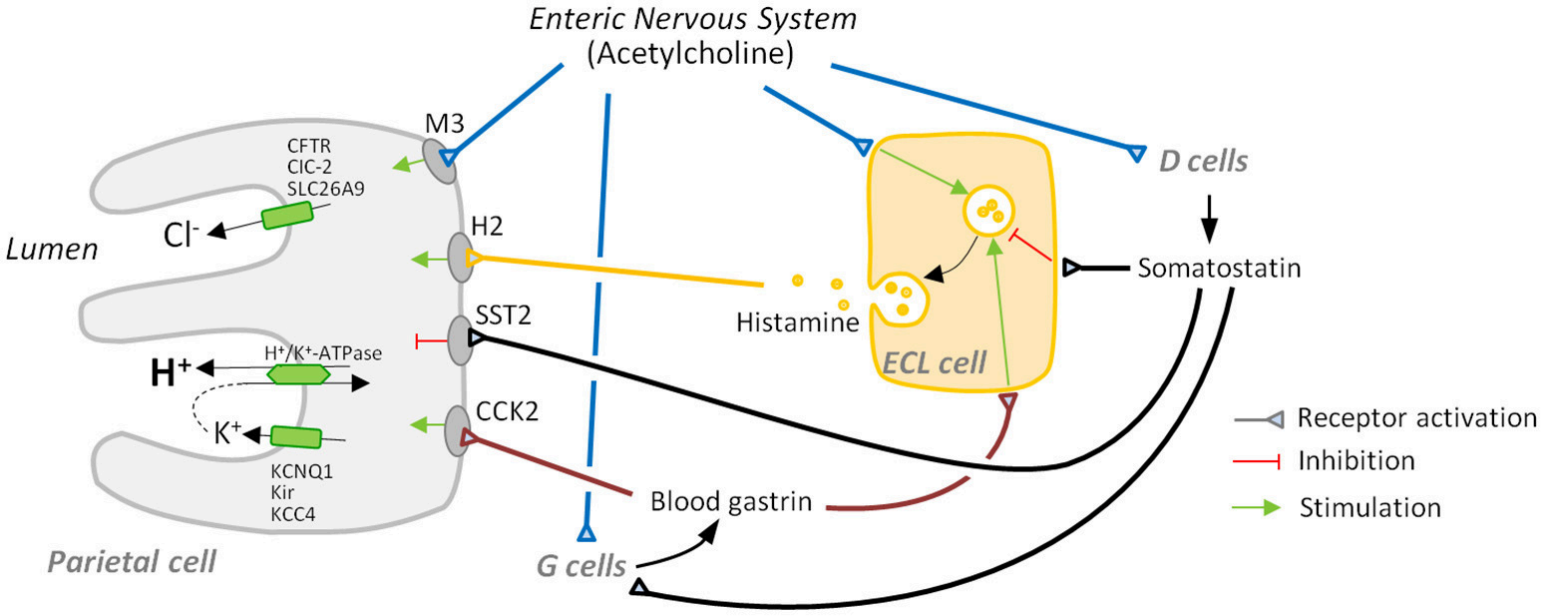
Major players in gastric acid secretion. Apical secretion of hydrochloric acid by the parietal cell requires three ion transport processes: an H^+^/K^+^-ATPase-mediated exchange of intracellular hydrogen ion for an extracellular potassium ion, and chloride secretion and potassium recycling, which are necessary to maintain electroneutrality and avoid luminal potassium depletion. Secretion is mainly regulated by activation: (i) of receptors for the secretagogues acetylcholine, histamine, and gastrin that are mainly secreted by the enteric nervous system, enterochromaffin-like (ECL) cells, and G cells, respectively, and (ii) of receptors for inhibitory somatostatin released by D cells.

Secretion of acid is regulated by an intricate network of paracrine (histamine, somatostatin), endocrine (gastrin, somatostatin), and neural [acetylcholine (ACh) and others] components. It involves the intercommunication of parietal cells with specialized cells of the gastric mucosa (ECL cells in the body and fundus, G cells in the antrum and D cells in the antrum, body, and fundus) and neurons (a comprehensive summary is shown in Table 1 and Figure 1).

**Table 1:** Functional specialization of the gastric gland cells.

**Cell type**	**Main location**	**Main stimulant**	**Substance secreted**	**Function of the secretion**
Mucous neck cells	Fundus	Tonic secretion	Mucus Bicarbonate	Provides a physic barrier between the lumen and the epithelium. Buffers gastric acid to avoid epithelium damage.
Parietal cells	Oxyntic area	ACh, Gastrin, Histamine	Hydrochloric acid (HCl, gastric acid) Intrinsic factor	Activates pepsin, kills bacteria. Permits vitamin B_12_ absorption.
ECL cells	Corpus	ACh, Gastrin	Histamine	Stimulates gastric acid secretion.
Chief cells	Corpus	ACh, Secretin	Pepsinogen Gastric lipase	Digests proteins. Digests fats.
G cells	Antrum	ACh, Peptides, Aminoacids	Gastrin	Stimulates gastric acid secretion.
D cells	Oxyntic area	Acid in the stomach, Gastrin, Cholecystokinin	Somatostatin	Inhibits gastric acid secretion.
Mucous cells (gastric epithelium)	Antrum	Tonic secretion	Mucus Pepsinogen	Provides a physic barrier between the lumen and the epithelium. Digests proteins.

*ECL, enterochromaffin-like; ACh, acetylcholine*.

### Vagal stimulation

Vagal stimulation of acid secretion has long been known. Parietal cells receive direct vagal stimulation, usually mediated by cholinergic neurons of the enteric nervous system (ENS). ACh acts via muscarinic M3 receptors (Kajimura et al., 1992) and induces the mobilization of Ca^2+^ from cellular stores via phospholipase C activation and inositol triphosphate elevation (Chew and Brown, 1986; Wilkes et al., 1991). Calcium subsequently activates several kinases, like calcium/calmodulin-dependent protein kinase II (CaMKII) and protein kinase C (PKC). CaMKII has a stimulatory effect on acid secretion, early proven by the fact that its pharmacologic inhibition abolishes the cholinergic activation of parietal cell secretion (Tsunoda et al., 1992). The role of PKC in acid secretion is more complex due to the different roles different PKC isoforms play. While PKC-ε induces a rise in basal intracellular calcium levels, therefore sensitizing the cell to stimulation, PKC-α inhibits acid secretion by down-regulating CaMKII activity (Fahrmann et al., 2002).

### Gastrin

Gastrin is a peptide hormone produced by G cells, present in the gastric antrum. It is released in response to a variety of stimuli. Amino acids and amines in the gastric lumen can stimulate gastrin release via calcium-sensing receptor (DelValle et al., 1990; Goo et al., 2010), thus G cells can directly respond to the arrival of food. They also receive direct stimulation of ENS neurons, which release ACh and gastrin-releasing peptide after input from the vagus nerve (Madaus et al., 1990; Debas and Carvajal, 1994). On the other hand, paracrine somatostatin arriving from nearby D cells represents the most important inhibitory signal for gastrin secretion (Zavros et al., 2003).

Gastrin travels in the bloodstream and directly and indirectly promotes acid secretion by acting on ECL cells and parietal cells upon binding to cholecystokinin (CCK) receptor type 2 (CCK2) (Kulaksiz et al., 2000). ECL cells respond by secreting histamine (Hakanson and Liedberg, 1970), which potently induces acid secretion by parietal cells (see below). This activation cascade is usually named gastrin-histamine axis. Gastrin might induce H^+^/K^+^-ATPase activation directly on parietal cells (Hills et al., 1996), although less evidence seems to support this concept. An analysis of isolated parietal cells indicated that CCK2 activation by gastrin induces a rise of intracellular calcium (Cabero et al., 1992), which might induce translocation of H^+^/K^+^-ATPase to the apical membrane in a similar way to cholinergic induction.

### Histamine

Histamine is secreted by ECL cells and acts on adjacent parietal cells, probably being the most potent inducer of acid secretion. ECL cells release histamine in response to gastrin and neuronal signals. ENS neurons also secrete pituitary adenylate cyclase activating polypeptide (PACAP), a neuropeptide that binds to a surface receptor of ECL cells and induces histamine secretion (Sandvik et al., 2001). Histamine acts on parietal cells via H2 receptor, a GPCR that induces both a G_s_-dependent activation of adenylate cyclase and cAMP increase and a G_q_-dependent rise in calcium levels (Hill et al., 1997). As discussed earlier, calcium has a positive effect on acid secretion, and so does cAMP. It activates protein kinase A (PKA) which, in turn, triggers a phosphorylation cascade that activates several downstream effectors leading to the translocation of H^+^/K^+^-ATPase to the apical membrane (reviewed by Yao and Forte, 2003). Given the central role of histamine in acid secretion H2 receptor has become a pharmacologic target of interest; H2 antagonists have been developed that prevent gastroesophageal reflux disease, although other drugs like proton pump inhibitors have been proven to be more effective (Khan et al., 2007).

### Somatostatin

Somatostatin is the main negative regulator of acid secretion. It is a hormone and paracrine peptide produced in the stomach by D cells. These cells are present in the oxyntic mucosa, where they negatively regulate ECL and parietal cell function, and also in the antral mucosa, where they negatively regulate G cell function (Alumets et al., 1979; Kamoshida et al., 1999). The physiology and morphology of both cell populations is somewhat different.

D cells secrete somatostatin in response to several stimuli. One of them is gastrin, which induces somatostatin secretion (Zavros et al., 1998), which in turn inhibits gastrin secretion from G cells. Therefore, the gastrin-somatostatin axis constitutes a negative feedback mechanism that maintains gastrin levels and acid secretion under control. Another positive stimulus is CCK, a peptide hormone secreted by the small intestine I cells as a response to luminal lipids; by stimulating somatostatin secretion CCK inhibits acid secretion during intestinal digestion (Konturek et al., 1992).

Luminal pH is probably the most important inducer of somatostatin release. Antral D cells are often called open type, because they possess extensions that make contact with the luminal content (Lamberts et al., 1991). Although the exact molecular pathway by which D cells sense luminal pH has not been described yet, calcium-sensing receptor is a plausible candidate (Goo et al., 2010; Adriaenssens et al., 2015). Apart from the direct effect on D cells, spinal neurons have been proposed to mediate luminal pH-induced somatostatin release. Oxyntic D cells are called closed type and are not in contact with gastric lumen, whereby it is unlikely they participate in pH sensing.

Cholinergic signaling has also been described to regulate somatostatin release, although its action is different on antral and oxyntic D cells; cholinergic agonists induce somatostatin release in antral D cells via M3 receptor (Buchan et al., 1992) while they inhibit somatostatin release in oxyntic D cells (Chiba and Yamada, 1990). Other ENS neuropeptides that have been described to induce somatostatin release include the vasoactive intestinal peptide (Zdon et al., 1988) and PACAP (Li et al., 2000).

Somatostatin functions as an overall brake on acid secretion since it negatively regulates parietal cells, G cells and ECL cells via SST2 receptor. In parietal cells, somatostatin inhibits acid secretion (Wyatt et al., 1996), in part via a G_i_ protein-induced decrease of cAMP levels (Park et al., 1987). In ECL cells somatostatin reduces Ca^2+^ currents, preventing intracellular calcium elevation induced by gastrin and, thus, exocytosis of histamine (Bjorkqvist et al., 2005). The molecular pathway by which somatostatin inhibits G cells has not been elucidated yet, although direct contacts between D cells and G cells and control of G cell function by somatostatin were recognized early (Larsson et al., 1979).

### Ghrelin

Ghrelin is a peptide that defines the anatomical body of the human stomach (Choi et al., 2014) and seems to induce acid secretion by stimulating histamine production by ECL cells (Schubert, 2015). Other compounds have been also reported to affect directly or indirectly gastric acid secretion. The effect of compounds like interleukin-1β, neurotensin, nitric oxide, oxyntomodulin, secretin, and serotonin is most likely inhibitory, although it remains a matter of debate (reviewed by Kopic and Geibel, 2013).

## Extracellular adenosine: sources and receptors

### Enzymes and transporters modulating extracellular adenosine

*In vivo* the extracellular concentration of a signaling molecule depends on a balance between its formation, release, uptake, and degradation or transformation. The adenine nucleotides AMP, ADP, and ATP and the nucleoside adenosine are components of the purinergic signaling. In general, ATP and adenosine are the main purinergic effectors. They are present both inside cells and in the extracellular milieu and are released by intact, living cells by different means.

**Figure 2:**
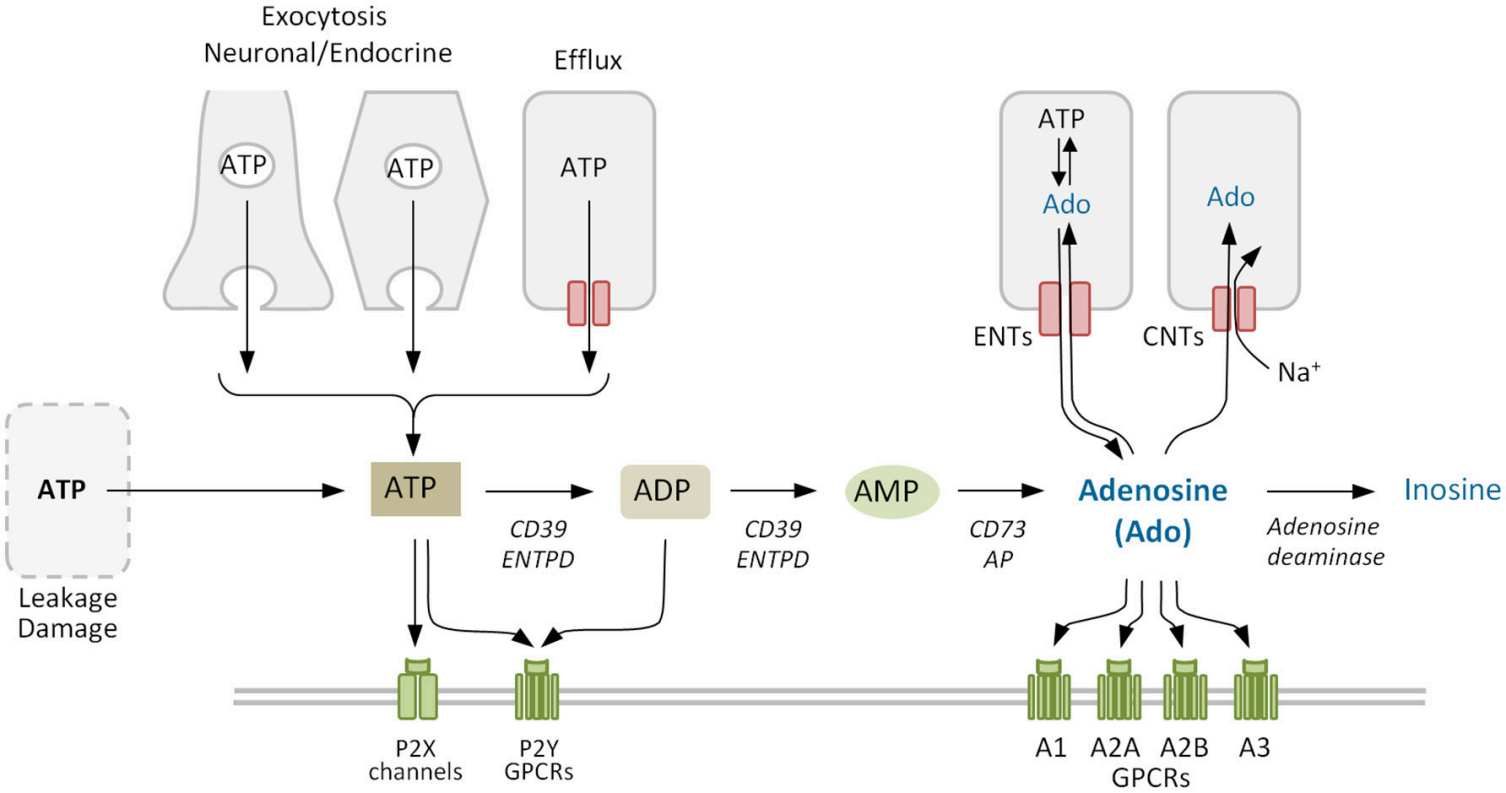
Adenosine metabolism in the extracellular space. Extracellular adenosine mainly derives from the phosphohydrolysis of precursor nucleotides ATP, ADP, and AMP by ectonucleotidases CD39 and CD73. Adenosine levels also depend on the activity of equilibrative and concentrative nucleoside transporters ENTs and CNTs, that allow the nucleoside to cross the plasma membrane, and of adenosine deaminase that degrades irreversibly adenosine to inosine. Purinergic receptors comprise the ligand-gated ion channel P2X and the G protein-coupled receptors (GPCRs) P2Y for nucleotides, and the four GPCRs for adenosine (P1). ENTPD, family of ectonucleoside triphosphate diphosphohydrolases. Purinergic receptors (P1 and/or P2) and other proteins co-expressed on the membrane may form cell type-specific combinatorial signaling units.

In neuroendocrine and exocrine cells ATP secretion occurs mainly via regulated exocytosis (Evans and Surprenant, 1992; Evans et al., 1992; Gualix et al., 1996; Sorensen and Novak, 2001; Lazarowski, 2012) (Figure 2). Under a variety of conditions ATP can also be transported through the plasma membrane by conductive mechanisms mediated by anion channels and by connexin hemichannels and pannexin channels (reviewed by Lazarowski, 2012). Concerning adenosine, it can be released through the ubiquitous equilibrative nucleoside transporters (ENTs; SLC29) following the concentration gradient (Griffith and Jarvis, 1996; Thorn and Jarvis, 1996). Increased levels of extracellular ATP can also lead to rapid formation of adenosine by the sequential action of the ectonucleoside triphosphate diphosphohydrolase (CD39) family of enzymes, which convert ATP to AMP, and ecto-5′-nucleotidase (CD73), which converts AMP to adenosine (Yegutkin, 2008).

As a signaling termination mechanism, adenosine is taken up by cells or metabolized in the extracellular medium (Figure 2). The uptake system includes ENTs, which are the main transporters in the rapid clearance of adenosine (Nguyen et al., 2015), and concentrative nucleoside transporters (CNTs), that move nucleosides against the concentration gradient (Thorn and Jarvis, 1996). Metabolization includes adenosine deaminase (ADA), which forms inosine as a terminal metabolite (Cristalli et al., 2001) and adenosine kinase, which regenerates the nucleotide AMP and thereby refills the adenine nucleotides reservoir (Boison, 2006). Using selective inhibitors Nguyen et al. (2015) demonstrated that multiple adenosine clearance mechanisms are redundant preventing adenosine extracellular accumulation.

### Release of ATP in the stomach wall

Most nerve terminals contain and release ATP together with classical transmitters such as ACh, norepinephrine, dopamine, glutamate, gamma-aminobutyric acid, and neuropeptides (Burnstock, 2013a, 2014b). This occurs in both the peripheral and the central nervous systems (CNS), although its relevance varies considerably in different species and pathophysiological conditions (Burnstock, 2013b, 2014b; Kennedy, 2015; King, 2015; Estevez-Herrera et al., 2016).

**Figure 3:**
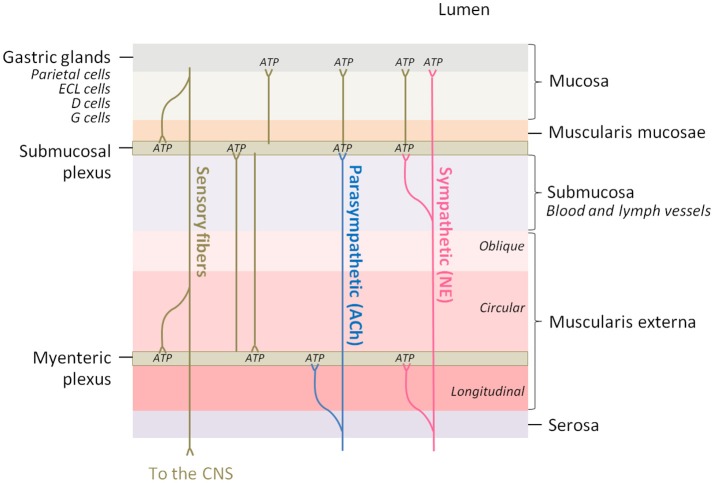
The innervation of the different layers of the stomach wall is a potential adenosine source. Parasympathetic fibers innervate the myenteric and submucosal plexuses of the enteric nervous system and acetylcholine (ACh) is the major neurotransmitter, while sympathetic fibers innervate the same regions and the gastric mucosa, being norepinephrine (NE) the major neurotransmitter. Brown lines represent innervation between different regions of the intrinsic nervous system. ATP, which is co-released in all the synaptic contacts, can be enzymatically transformed into adenosine.

In order to understand the functional implications that ATP release might have in nerve fibers innervating the different stomach structures it is useful to bear in mind the anatomy of the stomach wall (Figure 3). The ENS is the intrinsic nervous system of the gastrointestinal tract and is composed of the myenteric and the submucosal plexuses, which are integrated in the wall of the gastrointestinal tract. The ENS is connected to the CNS through sympathetic and parasympathetic nerves, and regulates several functions among which are included motility, glandular secretions, fluid transport, or local blood flow (Xue et al., 2016).

The stomach submucosal plexus presents virtually no ganglia, and so the abundant nerve fibers present in the oxyntic mucosa derive from the myenteric ganglia (Zhao and Chen, 2012). The majority of these neurons are cholinergic and are innervated by preganglionic efferents from the vagus nerve. Intact vagal innervation is crucial for gastric acid secretion (Kupari et al., 2013).

Purinoceptor expression is widespread in the gut. They are involved in synaptic transmission and neuromodulation in both myenteric and submucous plexuses and thus participate in secretion and motility (Burnstock, 2011, 2014a). ATP co-released in nerve endings can diffuse through the synaptic cleft and bind to post-junctional P2 receptors or be converted into adenosine by ectonucleotidases. In some cases, adenosine can act through P1 receptors in the prejunctional or post-junctional structures or in nearby cells.

For example, adenosine has been described to act as a modulator of neurotransmitter release. Adenosine regulates ATP and norepinephrine secretion in the prejunctional nerve terminals of the presynaptic neurons innervating smooth muscle (Burnstock, 2013a, 2014b). Adenosine also participates in the regulation of ACh release, which is a major excitatory neurotransmitter in the myenteric plexus. In the rat ileum ACh regulates its own release in an autocrine fashion by activating M3 muscarinic receptors present in the myenteric neuron, which in turn induces an outflow of endogenous adenosine. In a bimodal response, ATP would act first on P2 receptors inducing ACh and ATP co-release (Vieira et al., 2009) and the enzymatically generated adenosine would induce or reduce ACh release upon binding to A2A or A1 receptor, respectively (Duarte-Araujo et al., 2009). The fact that gastric parietal cells and D cells express A2B (Arin et al., 2015a,b) and A2A (Yip et al., 2004a), respectively, suggests that adenosine is a bona fide extracellular mediator in the neighborhood; it is possible, therefore, to speculate that the autocrine regulation described above for myenteric neurons might operate too in the cholinergic fibers of the ENS innervating both parietal and D cells, representing an additional regulatory mechanism for adenosine in gastric acid secretion.

The presence of ectonucleoside triphosphate diphosphohydrolase activity acting on ATP and ADP was demonstrated by Savegnano et al. in rat gastric mucosa. This hydrolase, besides participating in the extracellular metabolism of nucleotides, controls the gastric secretion of acid, pepsin, and mucus, as well as the stomach contractility (Savegnago et al., 2005). High levels of ATP diphosphohydrolase were also detected in the guinea pig stomach associated with parietal and chief cells (Sevigny et al., 1998) and ATPase activity was described in the stomach of mice and guinea pigs, in the neurons of myenteric and submucosal plexuses, in the muscular layer and mucosal nerve terminals (Lavoie et al., 2011). Those enzyme activities, by degrading ATP, participate directly in the extracellular adenosine availability. Adverse conditions, including hypoxia or inflammation, are associated with increased intracellular and extracellular dephosphorylation of ATP to adenosine through ectonucleotidases (Antonioli et al., 2008).

### Adenosine receptors

Extracellular adenosine exerts its action by interacting with four members of the large family of seven transmembrane GPCRs denoted A1, A2A, A2B, and A3 (Fredholm et al., 2011; Gessi et al., 2011; von Kugelgen and Harden, 2011). Significant advances have been made in our understanding of the pharmacological profile and function of these receptors as well as their molecular cloning, expression, and structure (reviewed by Trincavelli et al., 2010). Adenosine receptors are widespread throughout the body and seem to be involved in neurological and cardiovascular diseases, inflammation, and cancer, among other diseases and conditions (Borea et al., 2016). Major differences in the receptor subtypes are their affinities for the endogenous ligand adenosine (Fredholm et al., 2001b), internalization and desensitization (Klaasse et al., 2008), and recruitment of G proteins and activation of signaling cascades (Table 2). As an example, most of the effects evoked by A2A and A2B receptors are due to activation of adenylate cyclase, generation of cAMP, and activation of PKA, whereas A1 and A3 are associated with adenylate cyclase inhibition through pertussis toxin sensitive G_αi_ (Abbracchio and Burnstock, 1994). Regarding the potency of adenosine at these receptors, both agonist binding affinity and functional studies revealed that A1, A2A, and A3 sites show similar high affinities for adenosine whereas much higher concentrations of adenosine are required to activate A2B (Table 2) (Fredholm et al., 2001b, 2011; Yan et al., 2003).

**Table 2:** Receptors for adenosine are metabotropic P1 purinergic receptors.

**Type**	**Subtype**	**Main coupling/signaling via**	**Endogenous agonist**	K_i_ (nM, human)^*^ Agonist: Adenosine	K_i_ (nM, human)^*^ Agonist: NECA
P1	A1	Pertussis toxin-sensitive G_αi_ proteins ↓ Adenylate cyclase ↑ PLC—↑ IP_3_—↑ [Ca^2+^]i—PKC	Adenosine	100	14
	A2A	Cholera toxin-sensitive G_αs_ proteins ↑ Adenylate cyclase—↑ cAMP—PKA	Adenosine	310	20
	A2B	G_αs_ proteins and G_q_ proteins in some cells ↑ Adenylate cyclase—↑ cAMP—PKA ↑ PLC—↑ IP_3_—↑ [Ca^2+^]i—PKC	Adenosine	15,000	140
	A3	G_i_ proteins ↓ Adenylate cyclase ↑ PLC—↑ IP_3_—↑ [Ca^2+^]i–PKC—	Adenosine	290	25
P2	P2X1-7 (7 members)	Ligand-gated channels selective for monovalent and divalent cations	ATP		
	P2Y1-14 (8 members)	G proteins ↑ PLC—↑ IP_3_—↑ [Ca^2+^]i—PKC ↑ PLA_2_—↑ Arachidonic acid	ATP, ADP, UTP, UDP		

*IP_3_, inositol triphosphate; NECA, 5′-N-ethylcarboxamidoadenosine (a non-selective agonist for P1 receptors); PKA, protein kinase A; PKC, protein kinase C; PLC, phospholipase C*.

* *K_i_ values extracted from Yan et al. (2003)*.

While receptors for adenosine are P1 purinergic receptors, extracellular nucleotides activate P2 purinergic receptors (Abbracchio and Burnstock, 1994). P2 receptors fall into two families P2X (ionotropic) and P2Y (metabotropic) (Table 2), each one composed by several members (Burnstock, 2007). In general, P2Y receptors are GPCR and P2X receptors regulate cell function by opening ion channels selective for monovalent and divalent cations and neither G proteins nor effector enzymes appear to be directly involved. However, P2X may also regulate the levels of second messengers. As a matter of fact, there is a significant crosstalk between all purinergic networks, which superimposes difficulties in identifying the receptor and mechanism responsible for a certain downstream response.

If extracellular adenosine plays a physiological role or is involved in a pathological process, it must be present at effective extracellular concentrations. In healthy, unstressed tissues adenosine levels in the extracellular space are low due to its rapid metabolism and uptake. The basal level has been estimated to be in the 10^−8^–10^−7^ M range (Ballarin et al., 1991). Slightly higher levels (10^−6^ M) have been measured in the CNS (Hagberg et al., 1987). This would be sufficient to activate A1, A2A, and A3 receptors (Table 2) provided that these proteins are expressed on the cell surface at a certain density (Fredholm et al., 2001b; Yan et al., 2003). Under stress conditions, such as hypoxia, or other conditions leading to depressed cellular energy states, there is an acute increase in adenosine generation that can exceed the removal capacity, resulting in markedly increased extracellular adenosine concentrations. Release from cells with damaged cell membranes or necrotic cells could provide even larger increases in extracellular adenine nucleotides as intracellular ATP levels are typically 3–5 mM. This direct release seems to occur during pathologies such as oxygen-glucose deprivation or ischemia (Frenguelli et al., 2007). It has been reported that the local adenosine level increases 10-fold during hypoxia and 100- to 1,000-fold in ischemia (Zetterström et al., 1982; Hagberg et al., 1987; Dux et al., 1990; Ballarin et al., 1991), which would allow also cell responses mediated via A2B receptors in these settings.

Not only the levels of extracellular adenosine but also the expression of adenosine receptors is regulated in cellular stress (Murphree et al., 2005). In particular, hypoxia can stimulate the expression of the A2B receptor as its gene bears a hypoxia-inducible factor-1 response element in its promoter (Kong et al., 2006). Low oxygen tension in areas of tissues with poor blood supply stimulates the formation of hypoxia-inducible factor-1. Having in mind that this transcription factor is a central regulator of oxygen homeostasis and that it has been implicated in transcriptional regulation of anti-inflammatory or tissue-protecting signaling (Hart et al., 2011; Eltzschig et al., 2012), it is tempting to speculate that increased extracellular adenosine and parietal cell A2B expression (Arin et al., 2015b) may also be implicated in stomach protection from damage.

As a general rule, the higher the number of receptors the more potent the response to the agonist will be. In the particular case of adenosine receptors, being coupled to more than one G protein and signaling pathway, an increase in receptor number does not necessarily alter the maximal response; instead, it will shift the dose-response curve (Fredholm et al., 2011). To add more complexity, purinergic signaling might depend not only on the expression levels of individual membrane receptors but also on the combinatorial networks of receptors and other proteins coexpressed in a cell. Based among others on energy transfer and immunoprecipitation techniques, a variety of homo- and hetero-oligomers of P1 (A1, A2A, and A3) and P2Y subtype receptors have been described (Ciruela et al., 2006; Schicker et al., 2009; Hill et al., 2014). The tight interaction between A1 and A2A receptors, and G_i_ and G_s_ proteins described in a recent work (Navarro et al., 2016) is an example of this kind of oligomerization that could be part of a complex transducing mechanism capable of switching biological functions depending on the extracellular concentration of adenosine. Interactions between specific membrane receptors and enzymes involved in the extracellular metabolism of adenine nucleotides like CD37 (Schicker et al., 2009) or nucleosides like ADA (Ciruela et al., 1996; Franco et al., 1997; Gracia et al., 2008, 2013) could also have functional significance by modulating local agonist concentration and/or ligand binding to adenosine receptors.

### Adenosine effects on gastric acid secretion modulation

Over the years, a link between adenosine and gastric acid secretion has been suggested by a host of studies addressing the contribution of adenosine and specific receptors to gastric acid secretion in different model scenarios. The knowledge about the modulator role of adenosine on gastric acid secretion, however, may be yet defined as fragmentary and inconclusive. Nevertheless, it can be drawn that adenosine actions may be species-dependent, inhibitory or stimulatory, and direct and indirect.

Gastric acid secretion requires a complex network of interactions between secretagogues and inhibitory mechanisms involving endocrine, paracrine, and neural stimuli (see section Acid Secretion and Its Regulation). Hence, for a correct interpretation of the experimental findings about the effects of adenosine on acid secretion, the limitations of and the specific interactions occurring in each experimental protocol must be taken into consideration. In the studies in intact animals, findings reflect the plasticity of gastric acid regulatory mechanisms, such as neural stimulation, and the compensation by modulatory agents other than adenosine. In the perfused stomach, the effects observed mostly derive from the intrinsic mechanisms of control of the stomach. In the studies using the gastric mucosa, the secretory function of the parietal cell will depend of the mechanisms that the parietal cell itself has, in addition to the activatory (ECL cells and G cells) and inhibitory (D cells) influence of the other cells that are also homed by the gastric glands. And finally, in the studies with isolated parietal cells, the acid secretion rate will be defined by the integrity of a collection of intrinsic processes, including those that make and secrete the hydrochloric acid, the quality and quantity of adenosine receptors and the activity of the signaling machinery.

To facilitate comparisons and comprehend causes for seemingly potential discrepancies, we review here the most relevant publications clustered in five sections: studies in intact animals, isolated stomach, gastric mucosa, isolated gastric glands, and isolated gastric parietal cells. A summary of the experimental evidences is offered in Table 3.

**Table 3:** Adenosine actions on gastric acid secretion in human and animal models.

**Model**	**Species**		**Effector/treatment**	**Mechanism**	**Finding**	**Acid secretion**	**References**
Intact animal	Rat	Conscious—vagotomized	Ado	–	Decrease	Decrease	Puurunen and Huttunen, 1988
		Anesthetized—intact vagus	Ado	Vagal stimulation (P1)	Decrease	Decrease	Puurunen and Huttunen, 1988
		Unanesthetized	Ado analogs	Gastric volume	Decrease	Decrease	Westerberg and Geiger, 1989
		Unanesthetized	Ado	G_i_ coupled A1	Decrease	Decrease	Scarpignato et al., 1987
		Anesthetized	Ado analogs	Vagal stimulation	Increase	Increase	Puurunen et al., 1986
		Conscious	Ado analogs	Vagal stimulation	Decrease	Decrease	Glavin et al., 1987
Perfused stomach	Rat	–	Ado	–	Decrease	Decrease	Gandarias et al., 1985
		–	Ado	Gastrin-G cell (A1)	Decrease	Decrease	Yip et al., 2004b
		–	Ado	Somatostatin-D cell (A2A)	Increase	Decrease	Yip and Kwok, 2004
	Mouse	WT	Ado	Somatostatin-D cell (A2A)	Increase	Decrease	Yang et al., 2009
		A2A-KO	Ado > 1 μM	Somatostatin-D cells (A2A)	Increase	Decrease	Yang et al., 2009
		A2A-KO	Ado < 10 nM	Somatostatin-D cells (A1)	Decrease	Increase	Yang et al., 2009
		WT	ADA inhibition	Ghrelin (A2A)	Increase	Increase	Yang et al., 2011
		A1-KO	ADA inhibition	Ghrelin	Increase	Increase	Yang et al., 2011
		A2A-KO	ADA inhibition	Ghrelin	No effect	No effect	Yang et al., 2011
Gastric mucosa	Human	Antrum, hyperchlorhydria	Hyperchoridria	ADA activity	Increase	Decrease	Namiot et al., 1990
		Gastric ulcer	Ranitidine	ADA activity	Increase	Decrease	Namiot et al., 1991
		Antrum, *H. pylori* infection	Inflammation	ADA activity	No effect	No effect	Bulbuloglu et al., 2005
		Gastric ulcer	Infection	ADA activity	No effect	No effect	Namiot et al., 2003
Gastric glands	Rabbit	Corpus—basal HCl secretion	Ado and Ado analogs	–	Increase	Increase	Ainz et al., 1989
		Corpus—histamine—stimulated	Ado and Ado analogs	–	Increase	Increase	Ainz et al., 1989
		Corpus	Ado and Ado analogs	P1 purinoceptors	Increase	Increase	Gil-Rodrigo et al., 1990
Parietal cells	Dog	Basal HCl secretion	Ado	–	Decrease	Decrease	Gerber et al., 1985
		Histamine-stimulated	Ado	A1	Decrease	Decrease	Gerber and Payne, 1988
		Antrum	Ado	Gastrin-G cells (A1)	Decrease	Decrease	Schepp et al., 1990
		Antrum	Ado	Gastrin-G cells (A2)	Increase	Increase	Schepp et al., 1990
	Guinea pig	–	Ado	–	Decrease	Decrease	Heldsinger et al., 1986
	Rat	–	Ado and Ado analogs	–	No effect	No effect	Puurunen et al., 1987
	Rabbit	Corpus	Ado and Ado analogs	cAMP increase (A2)	Increase	Increase	Ota et al., 1989
		Basal and histamine—stimulated	Ado and Ado analogs	P1 (A2) receptors	Increase	Increase	Ainz et al., 1993
		Corpus	ADA treatment	–	Decrease	Increase	Arin et al., 2015b
		Corpus	Ado and Ado analogs	G_s_ coupled (A2B)	Increase	Increase	Arin et al., 2015b

*The “acid secretion” column denotes either the reported primary effect or the secondary effect that is deduced from the “Finding” according to current knowledge. Ado, adenosine; ADA, adenosine deaminase; KO, knockout; WT, wild-type*.

### Studies in intact animals

While dogs and rabbits were the preferred models in early *in vivo* studies on purinergic control of gastric acid secretion, most work aiming at establishing whether adenosine has a role in gastric acid secretion was performed in rodents. The administration of adenosine and its analogs was shown to inhibit gastric acid secretion in various species. However, the site at which adenosine acts seems to differ.

In conscious rats subjected to vagotomy or in anesthetized rats with intact vagal innervation, intracerebroventricular administration of adenosine inhibited gastric secretion in a dose-dependent manner. In the latter case, the inhibition was found to be due to a reduction in vagal efferent activity to the stomach acting at the brain level on xanthine-insensitive P1 receptors (Puurunen and Huttunen, 1988). These findings are compatible with other studies also conducted in unanesthetized rats, such as those of Westerberg and Geiger who demonstrated that adenosine analogs not only regulate the acidity but also the volume of gastric secretions; they showed that 5′-N-ethylcarboxamideadenosine (NECA) and 2-chloroadenosine decreased basal acid output in a dose-dependent manner and that low doses of NECA inhibited gastric volume almost entirely (Westerberg and Geiger, 1989). Also subcutaneous administration of adenosine to awake rats promoted an inhibitory effect that was mediated by adenosine binding to A1 receptor (Scarpignato et al., 1987). By contrast, it was observed that adenosine and related analogs increased gastric acid secretion in anesthetized rats after intravenous injection in an action that was totally prevented by vagotomy, suggesting that adenosine derivatives stimulate gastric acid secretion in anesthetized rats by activating some unidentified adenosine receptors harbored in the afferent via of the vagus nerve (Puurunen et al., 1986). Using a gastric cannula designed as a real-time H^+^ sensor, Glavin et al. (1987) observed that the ADA-resistant analog of adenosine R-phenylisopropyladenosine (R-PIA) led to a decreased acid secretion in conscious rats with intact vagal stimulation, while the P1 receptor antagonist 8-phenylteophylline augmented gastric acid output. These two studies strongly suggested that adenosine signaling pathways may have a secretory action in the rat when the connection vagus/ENS is properly integrated, which clashes with the other studies above.

Globally, these findings indicate that, *in vivo*, the functional status of vagal innervation and its proper integration into the ENS may determine the gastric response to adenosine, with contradictory results yet unresolved. It must be taken into account that the stomach is one of the most complex glandular organs in the body, that vagal innervation may account for up to 85% of the basal and 50–60% of the post-prandial secretion of acid (Debas and Carvajal, 1994), and that, as shown in Figure 1, the vagus not only acts directly on the acid-secreting parietal cell but also on the histamine-, somatostatin-, and gastrin-secreting cells of the gastric glands (Debas and Carvajal, 1994; Kopic and Geibel, 2013). Hence, differences in the effective concentration of extracellular adenosine as consequence of differences in the experimental design (such as in the administration route) might lead to apparently inconsistent responses.

Works on the phenotype of knockout mice for specific adenosine receptors have focused on inflammation, anxiety, vascular resistance, or tolerance to hypoxia/ischemia, to name some (reviewed by Fredholm, 2007), but gastric acid secretion disorders have not been reported. Research performed in the A2A receptor- and A1 receptor-knockout mouse stomach regarding acid secretion is detailed below.

### Studies in isolated perfused stomach

Rodents are also the preferred species for most experimentation on acid secretion using the whole stomach after “*in situ*” vascular perfusion with an isotonic saline solution. As a general rule, the perfused stomach maintains the chemical and neural interconnections of the gastric gland cells though the extrinsic secretomotor innervation (the vegetative system) may be attenuated by the unavoidable anesthesia.

A pioneer study by Gandarias et al. (1985) reported that adenosine (10^−3^–10^−4^ M) reduced the basal secretion of acid in isolated rat stomach whereas ATP, ADP, and AMP were able to elevate basal acid secretion dose-dependently. Notably, in the presence of adrenergic and cholinergic blockers like ergotamine, propranolol, or atropine, all purine derivatives, including adenosine, caused a significant increase in the basal acid secretion. Several further works evidenced that the adenosine-promoted reduction of HCl secretion in the rat stomach might be an indirect effect due to its binding to inhibitory A1 receptor in the gastrin-secreting G cells, leading to a reduction in the gastrin concentration in the perfusate and consequently in gastric acid secretion (Yip et al., 2004b). In the same line of thinking, it was also shown that the decrease in gastrin secretion might well be due to adenosine binding to the A2A receptor expressed in the gastric plexus and at the somatostatin-secreting D cells. Concomitant to the rise in somatostatin release, a rise in gastrin levels would occur as a counter-regulation loop which would increase acid secretion afterwards (Yip and Kwok, 2004). Findings supporting this view were reported by Schepp et al. (1990) in primary cultures of dog antral G cells, who found that gastrin secretion was inhibited by adenosine in a pertussis toxin-sensitive process involving A1 binding and activated by adenosine and related analogs in a cAMP-independent process involving A2 receptor.

Meanwhile, in experiments performed in isolated perfused stomachs of mice bearing genetic ablation of A2A receptor, adenosine was demonstrated to exhibit differential dose-dependent effects on acid secretion, so that at high concentrations (>1 μM), adenosine acted through A2A stimulating somatostatin secretion but when adenosine concentrations were below 10 nM it acted mainly through its inhibitory A1 receptor (Yang et al., 2009) decreasing somatostatin production. Theoretically, high adenosine would result in decreasing and low adenosine in increasing acid secretion.

Using pharmacological approaches in the A1 and A2A receptor knockout mice stomach, Yang and colleagues examined gastric ghrelin release. As mentioned in section Acid Secretion and Its Regulation, ghrelin seems to induce acid secretion by stimulating histamine production by ECL cells (Schubert, 2015). These authors found that adenosine exerts predominantly a tonic A2A receptor-mediated stimulatory action on ghrelin release, whereas an A1-mediated inhibitory action is also apparent when the tonic excitatory effect was blocked with tetrodotoxin. They also demonstrated that ghrelin release became activated by including an ADA activity inhibitor in the perfusate in wild type and A1 knockout mice but not in A2A knockouts, reinforcing the concept that circulating ghrelin and adenosine increase in parallel via A2A in mice (Yang et al., 2011). Whether this stimulatory axis operates in humans is an open question that deserves to be addressed.

### Studies in gastric mucosa

The purinergic regulation of acid secretion in gastric mucosa was first addressed by Kidder (1973) when purinergic receptors had not been yet discovered. Kidder demonstrated that ADP and ATP inhibited acid secretion in bullfrog gastric mucosa when added to the bathing saline solution.

Of particular relevance are the studies conducted in human gastric mucosa biopsies from healthy patients and patients affected by a gastric pathology. Unfortunately, the direct impact of adenosine on the secretory function of the gastric parietal cell or gastric mucosa glands has not been addressed, though numerous studies reporting a role for ADA activity in gastric acid secretion have been conducted. As ADA inactivates extracellular adenosine to inosine and inosine has a 7-fold lower affinity for adenosine receptors than the natural ligand or even no affinity (Fredholm et al., 2001b), an increase in ADA activity results in reduced adenosine signaling.

Namiot et al. (1990) measured ADA activity in mucosa samples taken endoscopically from the fundus and antrum areas of the stomach in patients having a normal acid secretion, achlorhydria, or gastric acid hypersecretion. They found that ADA activity was higher in the fundus than in the antral region, and that patients with hypersecretion exhibited the highest ADA activity in the fundic mucosa, correlating positively ADA activity and basal or maximal acid output. These findings led the authors to propose that ADA activity and, therefore, adenosine is another potential compound involved in the modulation of gastric acid secretion.

Biopsies of the antral mucosa of *Helicobacter pylori*-infected patients showed high ADA activity. However, a correlation of ADA activity with the degree of inflammation could not be established (Bulbuloglu et al., 2005). On their hand, Namiot et al. (2003) postulated that ADA activity intervened in the inflammatory response of the gastric mucosa to other stimuli but not to *H. pylori* infection. In that study, ADA activity was measured in biopsies collected from patients infected with *H. pylori* that had developed chronic gastritis and that had been submitted or not to distal resection, as well as non-infected controls. Findings demonstrated that ADA activity in partially resected stomachs was lower than in intact stomachs and revealed that infection had no effect on ADA activity. Based on this, the authors concluded that ADA activity does not seem to be a factor promoting chronic gastritis. Adenosine has been defined as an endogenous anti-inflammatory agent released by cells in metabolically unfavorable conditions (Ye and Rajendran, 2009; Colgan and Eltzschig, 2012; Borea et al., 2016), but the studies above reported indicate that such conclusion should not be straightforwardly extrapolated to gastric mucosa.

In gastric ulcer patients, ADA activity in the mucosal body of the stomach was stimulated by ranitidine (an H_2_ receptor blocker) treatment (Namiot et al., 1991). These studies, therefore, suggest that adenosine contributes to inhibit gastric acid secretion and indirectly acts as a gastroprotective agent. Similarly, in animal studies, adenosine and its analogs have been shown to protect against stress-induced gastric ulcer formation (Geiger and Glavin, 1985; Westerberg and Geiger, 1987).

Besides, adenosine has been suggested to have anticancer effects on gastric cancer cells (Geiger and Glavin, 1985; Westerberg and Geiger, 1987). In addition to other proposed mechanisms, such as those promoting apoptosis of gastric cancer cells acting through intrinsic and extrinsic signaling pathways (Wang and Ren, 2006; Tsuchiya and Nishizaki, 2015), the inhibitory adenosine effect on gastrin secretion might be of relevance (Yip et al., 2004b). As mentioned in section Acid Secretion and Its Regulation, the hormone gastrin stimulates acid secretion acting directly on the parietal cell and indirectly by activating the histamine-producing ECL cells. There are many arguments in favor of a role of gastrin and its target cell, the ECL cell, in gastric carcinogenesis. Thus, not only the function but also the proliferation of ECL cells in the stomach is regulated by gastrin (Smith et al., 2017; Waldum et al., 2017).

Durak et al. (1994) showed that mucosal ADA activity was markedly elevated in patients with gastric cancer, which led the authors to suggest that this elevated rate of extracellular adenosine degradation may be behind the accelerated nucleotide metabolism of gastric cancerous tissues as compared with the normal tissue. Given that the higher ADA activity in cancer tissue might underlie the decreased adenosine concentration (Durak et al., 1994), higher escape from apoptosis might be occurring in gastric cancer because of low extracellular adenosine levels. Interestingly, this is compatible with the theory that it is mainly atrophic gastritis of the oxyntic mucosa that predisposes to gastric cancer possibly by inducing hypoacidity and hypergastrinemia (Waldum et al., 2017).

### Studies in isolated gastric glands

Two studies attempted to characterize whether purinergic signaling regulates acid secretion using gastric glands isolated by enzymatic digestion from the rabbit corpus mucosa. The authors presented evidence indicating that in non-stimulated gastric glands adenosine was able to increase acid secretion in a dose-dependent manner, whereas the adenine nucleotides AMP, ADP, and ATP did not produce any response (Ainz et al., 1989). After histamine stimulation glands behaved in a different way, and both AMP and adenosine had a synergistic stimulatory effect on HCl secretion whereas ADP and ATP induced graded inhibition of the histamine-promoted activation of acid secretion. The stimulatory action of adenosine was confirmed in a later work that reported that adenosine and ATP had opposing effects on acid secretion in both histamine-stimulated and unstimulated glands with a positive effect for adenosine and negative for ATP and ADP (Gil-Rodrigo et al., 1990). The fact that theophylline abrogated the stimulatory action of adenosine and that indomethacin, an inhibitor of prostaglandin synthesis, reduced the inhibitory response of ATP, led the authors to conclude that purinergic compounds are important modulators of gastric acid secretion and that the stimulatory responses may be mediated by P1 purinoceptors whereas the inhibitory responses may be mediated by P2 purinoceptors.

### Studies in parietal cells isolated from gastric mucosa

First studies on acid secretion using isolated parietal cells were performed in dogs by Gerber and colleagues in the 1980s (Gerber et al., 1985). They revealed the existence of inhibitory adenosine receptors of “Type R” on parietal cells and that adenosine inhibited directly gastric acid secretion. Further studies by the same group suggested the presence of A1 receptors involved in the inhibition of the histamine-stimulated acid secretion (Gerber and Payne, 1988). Since ADA addition resulted in an enhanced histamine-stimulated acid production, it was claimed that endogenous adenosine of canine parietal cells could modulate acid secretion by interaction with the receptors harbored on the parietal cell membranes. In the guinea pig parietal cell, Heldsinger et al. (1986) came to the same conclusion, although in this case the receptors involved were not identified.

As mentioned before, cAMP plays a role in several signaling pathways involved in acid secretion regulation in the gastric parietal cell. A2 receptors couple to stimulatory G_s_ proteins and activate adenylate cyclase and the formation of cAMP whereas A1 receptors act through G_i_ inhibiting cAMP formation upon activation. Ota et al. (1989) were the first to study the effects of adenosine and adenosine analogs on acid secretion in isolated rabbit parietal cells. For testing the changes promoted by these compounds, they measured the accumulation of [^14^C]aminopyrine in cells as an indicator of the acid that is trapped in intracellular compartments. The authors found that adenosine and related analogs caused proportional increases in [^14^C]aminopyrine accumulation as well as in cAMP concentration, suggesting the direct participation of adenosine in the regulation of gastric acid secretion mediated by A2 receptors (Ota et al., 1989). In the rat parietal cell, however, opposite effects were found, as adenosine did not affect at all [^14^C]aminopyrine uptake and cAMP levels (Puurunen et al., 1987). These findings may be interpreted to mean: (i) that the adenosine receptor expression and number in gastric parietal cells is species-specific or (ii) that a contamination of other cell types in the culture might influence the results. Also using isolated rabbit parietal cells, Ainz et al. (1993) proposed the existence of purinoceptors P1 (A2/Ra) that regulated the concentration-dependent stimulatory effects of adenosine, NECA and 2-chloroadenosine on gastric acid secretion in basal and dibutyryl-cAMP- or histamine-stimulated conditions. The response to NECA of parietal cell acid secretion was found to be blocked by theophylline, a non-specific P1 purinoceptor antagonist.

Using parietal cells isolated from rabbit gastric glands of the stomach corpus, we performed the pharmacological identification of A2B and demonstrated that degradation of endogenous adenosine secondary to ADA treatment reduced acid secretion (Arin et al., 2015b). The cells we used were representative populations of primary parietal cells at rest, thus suggesting that endogenous adenosine may contribute to spontaneous acid production in basal conditions. Furthermore, exposure of such cells to adenosine analogs stimulated acid secretion, and further pharmacologic and functional studies revealed that A2B was the only receptor involved (Arin et al., 2015b). Our study demonstrated that, in rabbits, the gastric parietal cell is endowed with a density of A2B receptors sufficient to promote acid secretion even though the affinity constants were similar to those reported in other tissues and cell models (Klotz et al., 1998; Fredholm et al., 2001b, 2011). We also demonstrated that such activation was mediated by a cAMP rise and not by calcium (Arin et al., 2015b). In human crypt epithelial cells, activation of A2B receptor by adenosine promotes Cl^−^ secretion via intracellular cAMP increase (Antonioli et al., 2008). The presence of the chloride channel CFTR at the apical pole of parietal cells has been confirmed in mouse (Sidani et al., 2007) and human stomach (Strong et al., 1994). As other components of the machinery of acid secretion CFTR is regulated by cAMP (Kopic and Geibel, 2013). It would be interesting to establish whether the regulation of acid secretion by A2B through cAMP is mediated by the modulation of CFTR.

Furthermore, flow cytometry and confocal microscopy revealed that ADA colocalized partially with A2B receptor on the parietal cell membrane and a link between the activities of these two proteins in the modulation of acid secretion was claimed (Arin et al., 2015a). A2B receptor was also found to have a vicinity relationship with ADA at the surface of the histamine-producing ECL cell (Arin et al., 2017). The physiological relevance, if any, of these findings remains to be elucidated. A2B signaling on distinct cell types and tissues is protective in conditions such as metabolic stress or during inflammation-associated tissue hypoxia or ischemia (Fredholm, 2007; Ye and Rajendran, 2009; Feoktistov and Biaggioni, 2011; Colgan and Eltzschig, 2012; Borea et al., 2016). Future studies should be done to carefully delineate if this protective effect is reproduced in the human stomach.

## Concluding remarks and future directions

There is a large body of evidence to implicate adenosine signaling in gastric acid secretion modulation. It is now recognized that adenosine influences acid secretion in a variety of direct and indirect ways. Findings are seemingly contradicting, but the scientific effort of the past years has permitted the identification of specific adenosine receptors and signaling pathways operating at the membrane of the parietal cells and other cells of the gastric mucosa in numerous species. From a physiological and fundamental point of view, this review has attempted to underline the complexity of the regulation of acid secretion.

The precise role adenosine has in the parietal cell function or the gastric gland physiology in humans is not clear. Although findings in the human gastric mucosa are the most attractive and important for obvious reasons, we cannot come to a conclusion yet. Studies addressed ADA activity in mucosal biopsies in patients with a diversity of pathologies. Adenosine is considered to have an anti-inflammatory action (Fredholm, 2007). However, in *H. pylori*-infected patients (Bulbuloglu et al., 2005) or in patients with chronic gastritis (Namiot et al., 2003), no correlation between ADA activity and mucosal inflammation was found. A positive correlation between ADA activity and basal and maximal gastric acid output was found in the fundic mucosa (Namiot et al., 1990), suggesting a protective, negative influence of adenosine on acid secretion from fundic parietal cells. However, considering the low proportion of H^+^/K^+^-ATPase-positive cells in the fundic area of the human stomach and that 95% of parietal cells were found within the oxyntic mucosa of the stomach (Choi et al., 2014), the physiologic relevance of these findings may be questioned. Given the differences between species, extrapolation of the findings in animal models to humans should be avoided. Another limitation comes from the fact that there are not non-transformed cells modeling those homed by the gastric glands. Much work is needed in this area. In keeping pace with the depth of knowledge of the mechanisms underlying acid secretion, it is necessary to ask more sophisticated questions to the cellular components of the human stomach.

Because adenosine receptors are widespread throughout the body and extracellular adenosine is a ubiquitous signaling molecule that modulates a wide variety of physiological processes and pathologies, it is generally believed that understanding how the different receptor subtypes are expressed and regulated in each cell type or functional module is a necessary step. The pleiotropic effects of adenosine exacerbate the dilemma of the drug/cell/tissue selectivity and pre-miRNAs and anti-miRNAs may have a chance of success. An exciting, recent discovery about purinergic signaling has been that A2A, A2B, and some P2X receptors, as well as some enzymes involved in extracellular adenosine metabolism, are subject to microRNA regulation (for a recent review, see Ferrari et al., 2016). Therefore, in theory, it may be possible to transiently modulate or permanently block the activation of a particular adenosine receptor subtype in a localized tissue using specific microRNAs or anti-microRNAs, which fosters the evaluation of microRNA technology-based treatments in purinergic network deregulation-associated diseases.

There is no doubt that extracellular ADA activity in the stomach plays a role in gastric acid secretion modulation and that adenosine receptors have been characterized in a host of cells of the gastric glands and ENS. But we must be aware of the fact that, though the presence of receptors for adenosine defines another potential compound involved in the modulation of gastric acid secretion, it does not define the importance of this compound during basal or stimulated acid secretion. The role of adenosine on gastric acid secretion requires clarification and great efforts should be done to define it further.

## Author contributions

RA, YR, OF, and BO conceived the study and wrote the manuscript. AG and HN-I contributed to the literature search. All authors approved the final manuscript.

### Conflict of interest statement

The authors declare that the research was conducted in the absence of any commercial or financial relationships that could be construed as a potential conflict of interest.

## Abbreviations

ACh: acetylcholine
ADA: adenosine deaminase
ADP: adenosine 5′-diphosphate
AMP: adenosine 5′-monophosphate
ATP: adenosine 5′-triphosphate
CaMKII: calcium/calmodulin-dependent protein kinase II
cAMP: cyclic adenosine 5′-monophosphate
CCK: cholecystokinin
CNS: central nervous system
CNT: concentrative nucleoside transporter
ECL: enterochromaffin-like
ENS: enteric nervous system
ENT: equilibrative nucleoside transporter
GPCR: G protein-coupled receptor
NECA: 5′-N-ethylcarboxamideadenosine
PACAP: pituitary adenylate cyclase activating polypeptide
PKA: protein kinase A
PKC: protein kinase C
SLC: solute carrier
SST: somatostatin
UDP: uridine 5′-diphosphate
UTP: uridine 5′-triphosphate.
